# Stronger Evaluations are Needed for Interventions to Improve Health Worker Performance

**DOI:** 10.4269/ajtmh.24-0539

**Published:** 2024-10-29

**Authors:** Alexander K. Rowe

**Affiliations:** Independent Consultant Atlanta, Georgia E-mail: samalexrowe@att.net

Dear Editor,

Outreach Training and Supportive Supervision (OTSS) is an intervention for improving health worker performance for malaria services.^1^ First piloted in 2009, it has been used in more than a dozen countries. For OTSS, supervisors are expected to visit health facilities at least once or twice each year, observe and record health worker practices with a checklist, and take corrective actions (e.g., on-the-job training, trouble-shooting, action planning, and timely follow up).^1^ Ashton et al. analyzed data from OTSS supervisory checklists from 2018–2021 in four African countries and concluded that their evaluation “provides strong evidence” that multiple OTSS visits improved malaria services.^2^ Although it is laudable to study interventions to improve health worker performance in low-resource settings, this particular evaluation had important limitations.

1) A large majority of the 5,119 OTSS-exposed facilities were excluded (4,635 facilities because they had received only one OTSS visit, 542 because of high baseline performance, and others because of missing values), which meant that the eight main analyses (Table 3 of the article) only included 1.2% (63/5,119) to 11.2% (572/5,119) of all OTSS-exposed facilities. As OTSS requires multiple facility visits, the first exclusion (facilities visited only once, despite a 4-year study period) is noteworthy because it shows that OTSS was usually not implemented as designed. Additionally, a facility’s receipt of multiple OTSS visits was not random: facilities might have been visited multiple times because they were performing poorly and needed extra support, because they were relatively easy to reach, or perhaps for other reasons. The article says little about why some but not other facilities received multiple visits. Thus, selection bias is plausible, and it is difficult to predict the direction and magnitude of this potential bias.
2) Besides recording health worker practices, OTSS requires supervisors to perform other tasks during facility visits, such as trouble-shooting and action planning. However, the article does not describe how often or how well these tasks were done (i.e., the intervention’s fidelity). Suboptimal fidelity can reduce an intervention’s effectiveness, and it is difficult to interpret an evaluation that lacks a description of how well the intervention was implemented.
3) The article does not describe any non-OTSS interventions (e.g., non-OTSS training or supervision, financial incentives, or digital interventions) or non-intervention events (e.g., medicine stock-outs, civil unrest, or COVID-19) that could have impacted upon OTSS implementation and study outcomes. These non-intervention exposures are potential confounders of an association between OTSS and performance outcomes.
4) The evaluation design was an uncontrolled pre-post intervention study with several measures in the post-intervention period. Commonly used frameworks (e.g., from the Cochrane Effective Practice and Organization of Care Group) consider the study’s observational design to have a high risk of bias,^3^ which essentially means a high likelihood that the true intervention effect could be markedly different from what the study found.
5) The study’s data were from checklists that supervisors completed during OTSS visits. Supervisors were trained to collect data with OTSS checklists, which is good; however, unlike gold-standard health facility surveys, there was no system to assess (and improve, if needed) data quality. Additionally, as explored in other health areas,^4^^,^^5^ there is a potential risk that real or perceived pressure to show improvement might have led some supervisors to report data with a positive bias. The article does not mention any data quality assessments. Thus, there are concerns about the validity of the study’s data.
6) The study analyzed continuous health worker performance indicators as dichotomous outcomes, and the only measures of OTSS effectiveness were odds ratios. The conversion of continuous indicators to binary ones makes it difficult to understand changes below the study’s 90% performance threshold (e.g., a large improvement in health worker practices from 0% to 89% would, for a dichotomized outcome, appear as no change at all). Additionally, as baseline values were not reported, it is difficult to interpret odds ratios in terms of absolute percentage-point changes. Absolute changes (presented with baseline values) are important because: a) they indicate the programmatic relevance of an intervention’s effects; b) they are more easily understood by decision-makers who lack statistical expertise; and c) they can be used to calculate other useful measures, such as cost-effectiveness, effective healthcare coverage, and impact.
7) The appropriateness of the logistic regression model is unclear. The model included up to five OTSS visits per facility; however, there are few data for higher order visits. Of 1,494 visits analyzed, only 6 (0.4%) were for fifth visits, Cameroon had zero fourth and fifth visits, and Ghana had zero third, fourth, and fifth visits. Thus, it seems questionable to make out-of-range predictions, which appear in all eight graphs in the article’s Figure 2. The data gaps raise concerns about the validity of the article’s main statistical model and predictions.

Given the above-mentioned methodological limitations and potential biases (several of which are mentioned by the authors), the article’s conclusion that its evidence of OTSS effectiveness is “strong” is a mischaracterization. As OTSS might truly be an effective intervention and given that it will likely be used in the future, a more rigorous evaluation of its effect on health worker performance is warranted. Another key objective of such an evaluation should be to understand whether or how multiple OTSS visits per facility can be delivered sustainably.
